# Supplementary material for: Towards culture-free sequencing of Mycobacterium tuberculosis: evaluating new targeted and whole-genome approaches for genotyping and drug resistance profiling
Source: Genome Med. 2026 Jul 21;18:152. doi: 10.1186/s13073-026-01726-7 (PMC13670635; doi:10.1186/s13073-026-01726-7)
Supplement: Supplementary file 1 [file 13073_2026_1726_MOESM1_ESM.docx]

**Additional file 1**

**Fig. S1**: **A.** Flowchart representing the method workflow for each sample analysed, including WGS from culture, tNGS (Deeplex XL Myc-TB) and dWGS (QIAseq xHYB MTB). **B.** Flowchart indicating the number of MTBC clinical sediments selected, analysed and interpreted for each study.

S1-A

S1-B

**Fig. S2: A, B.** qPCR quantification of MTBC genome copies (gc/µL): distribution of samples by concentration range

S2-A

S2-B

| **genome copies (gc)/µL** | **Cycles threshold (Ct) range** | **gc/µL mean** | **N samples** | **%** |
| --- | --- | --- | --- | --- |
| **<10** | 31-37 | 2.6 | 21 | 21.9 |
| **10-100** | 28-31 | 40.7 | 34 | 35.4 |
| **100-1000** | 24-27 | 330 | 29 | 30.2 |
| **>1000** | <24 | 4012 | 12 | 12.5 |

**Fig. S3:** Transmission Analysis **A.** MST illustrating clusters from dWGS, with MST cluster 1 highlighted in pink**; B.** MST illustrating clusters from cWGS, with the same MST cluster 1 highlighted in red. Cluster distance threshold = 5 alleles. Generated with core genome multi-locus sequence typing (cgMLST) scheme implemented in Ridom SeqSphere+ software v.10.0.5 (Ridom GmbH) (now MBioSEQ Ridom Typer, Bruker, Germany).

S3-A

S3-B

**Fig. S4:** Decision algorithm for implementation**,** bacillary-load–guided sequencing strategy for DR-TB diagnosis.
